# Perceptions of recurrence risk and behavioural changes among first‐ever and recurrent stroke survivors: A qualitative analysis

**DOI:** 10.1111/hex.13335

**Published:** 2021-08-06

**Authors:** Beilei Lin, Zhenxiang Zhang, Yunfei Guo, Wenna Wang, Yongxia Mei, Shanshan Wang, Yao Tong, Nazia Shuaib, Daphne Cheung

**Affiliations:** ^1^ Nursing and Health School Zhengzhou University Zhengzhou Henan PR China; ^2^ Academy of Medical Sciences Zhengzhou University Zhengzhou Henan PR China; ^3^ School of Nursing Hong Kong Polytechnic University Kowloon Hong Kong PR China

**Keywords:** healthy behaviour, perception, qualitative, recurrent risk, stroke

## Abstract

**Background:**

Among stroke survivors, the risk of stroke recurrence is high, and stroke survivors' perception of the risk of recurrence is crucial to promote healthy behaviours.

**Objectives:**

This study aimed to explore the perceptions of stroke survivors about their risk of recurrence and healthy behavioural modifications.

**Design:**

A qualitative interview study was carried out.

**Results:**

We interviewed 19 stroke survivors from 3 hospitals. Thematic analysis showed that the perceptions of recurrence risk and healthy behavioural changes differed between first‐ever and recurrent stroke survivors. Three themes were generated from the data of first‐ever stroke survivors: indifference to and unawareness of the risk of stroke recurrence, the need for professional information support and different awareness of the importance of different healthy behaviours. For first‐relapse stroke patients: worry but feel powerlessness towards recurrent event, accurate information is still warranted, regret of unhealthy behaviour patterns. For the survivors suffered two or more times recurrences: perceived severity of recurrences, increased psychological care need, incorrect perceptions of healthy behaviour.

**Discussion and Conclusion:**

Stroke survivors with or without recurrence hold different perceptions towards the risk of recurrence and behavioural changes. The need for information related to warning signs, recurrence risk and risk factors remained consistently unmet. The benefits of healthy behaviours could be a double‐edged sword for the prevention of stroke recurrence if the survivors fail to understand these accurately. It is strongly recommended that a specific recurrence risk communication tool and related health education plan be explored on the basis of the number of times patients have experienced stroke recurrence to inform secondary prevention of stroke in the future.

**Patient/Public Contribution:**

The patients were involved in the formulation of interview questions and conduct of this study. No public was involved in this study.

## 1 INTRODUCTION

Despite many advances in stroke prevention and treatment, stroke is still the leading cause of death and disability worldwide.^1^ Also, patients who survive after an initial stroke are known to be at a significantly increased risk for stroke in the future.^2^ The risk of recurrence of stroke is 11.1%, 26.4%, 39.2% and 39.7% at 1, 5, 10 and 12 years, respectively,^3^, ^4^ and a significant proportion of all hospitalized stroke cases are due to recurrence.^5^ Fortunately, 80% of recurrent strokes can be prevented by addressing the modifiable risk factors; therefore, secondary prevention after the first stroke is very important.^6^ For secondary prevention of stroke, an understanding of the risk factors for recurrent stroke and the appropriate lifestyle modifications necessary to minimize risk is essential among stroke survivors.^7^ According to the health behaviour theory,^8^ accurate perceptions of risks to one's own health are critical to adopt changes in health‐relevant behaviours. A study conducted in the United Kingdom showed that stroke survivors who were aware of the risks to their health made significant lifestyle modifications after stroke, that is, decreased consumption of salt and increased consumption of vegetables or fruits.^9^ However, most stroke survivors do not have an accurate understanding of the risk of recurrence of stroke.^10^, ^11^, ^12^ Boden‐Albala et al.^12^ reported that only 20% of stroke survivors had an accurate understanding of their risk for a recurrent stroke; 10% of the survivors underestimated their risk and 70% significantly overestimated their risk. Another study conducted in Thailand revealed that most survivors wrongly estimated their risk of further stroke; 43.6% of survivors underestimated their risk and nearly one‐fifth (17.1%) overestimated their risk.^10^ In summary, quantitative evidence shows that overestimation or underestimation of recurrence risk is not unusual among stroke survivors.

Although a large number of qualitative studies focusing on patients with stroke have been conducted,^13^, ^14^, ^15^ very few quantitative studies have focused on the perception of recurrence risk among stroke survivors.^10^, ^11^, ^12^ Additionally, Hiraga^7^ reported that both knowledge and perception of recurrent stroke risk were crucial for secondary prevention of stroke; he also claimed that the perception of risk and overestimation may gradually decrease with time, and underestimation might increase after the first stroke. Wondergem et al.^16^ pointed out a significant difference in disease cognition, coping attitudes and behaviour adjustment in stroke survivors with first‐onset and recurrent events. Other scholars followed stroke survivors for 3 years and conducted in‐depth interviews to explore their life experience after stroke, and found that life after stroke always involved a continuous adjustment and adaptation process.^17^ Thus, this may raise the research question of whether individuals with new‐onset and recurrent stroke have different perceptions of their susceptibility to and risk of recurrent stroke. However, to date, this issue remains unclear.

Risk perception is an individual's capability to acknowledge the presence of a certain degree of risk; it refers to people's subjective judgements about the likelihood of negative occurrences such as injury, illness, disease and death.^18^ An understanding of patients' awareness of the risk of recurrent stroke is important and essential for translating the perception and knowledge of recurrent stroke risk and individual risk factors into appropriate behavioural changes.^7^ Ignoring the risk of recurrence may affect stroke survivors' attitude towards prognosis and response to secondary prevention. However, misperceptions of the risk of chronic diseases are common among the public, and the relationships between these beliefs and consequences are also complex.^19^ Therefore, using a qualitative research approach, this study aimed to provide insights into the perceptions of recurrence risk and healthy behavioural modifications among stroke survivors who experienced first‐ever and recurrent stroke. The results will then inform the development of interventions based on frequency of relapse to promote effective health education and stroke secondary prevention strategies.

## 2 METHODS

### 2.1 Setting and sample

This qualitative analysis forms the basis of an intervention study, which has been approved by the Chinese Clinical Trial Registry, that aims to provide a basic understanding of stroke survivors' perceptions of recurrence risk and behavioural modifications. Stroke survivors with first‐onset or recurrent events were recruited from two city‐level hospitals (city H) and one county‐level hospital across two cities in Henan, China (Zhengzhou city and Anyang city). The inclusion criteria were as follows: survivors of various types of stroke^20^ with communication ability (Token test ≥ 17 points)^21^ and self‐care capability (activities of daily living ≥ 40)^22^ and individuals without cognitive dysfunction (mini mental state examination ≥ 17 points).^23^

### 2.2 Data collection

The demographic information of the stroke survivors was collected using a short questionnaire. The preliminary interview guide was formulated based on explorative interviews and extensive literature reviews. A discussion within the research group was held, and three relevant experts in stroke care research, psychological care and behaviour management were identified and consulted to formulate the interview guide. The focus was on first‐ever and recurrent stroke survivors, including six questions related to their experience of stroke onset and opinions about the risk of recurrence (Supporting Information Appendix).

The interview was conducted from April 2019 to April 2020. The interviews were conducted in a quiet consultation room in hospitals by two researchers using a semi‐structured interview guide and audio recordings. The survivors were informed of the purpose and significance of the research, the possible interview duration and the recording methods. The interview was started after written consent was obtained from the survivors. A relaxed atmosphere was maintained, and interview techniques such as questioning, listening, response, follow‐up and repetition were used to encourage the interviewees to express their true views and feelings. Field notes were written immediately during or after the interview and included observations, reflections and nonverbal behaviours, such as tone, expression, eye contact and subtle body language of the interviewees. When no new topics emerged, a group discussion on data saturation was held to achieve consistency. In addition, the Essen stroke risk score was used in this study to estimate the stroke survivors' risk of recurrence^24^; the Barthel index was scored and the self‐care level of the interviewees was determined.^22^ During the data collection process, if the interviewees exhibited sadness, we discontinued the interview and comforted the participants until they felt better.

### 2.3 Data analysis

The recording was transcribed. After transcription, 4 out of 19 sets of verbatim responses were randomly selected for checking to ensure accuracy. The data were analysed inductively using thematic analysis strategies.^25^, ^26^ The analysis steps were as follows: (1) the interview transcripts were read and reread by two researchers to familiarize themselves with the data as a whole, and to make sense of the data, preliminary ideas were captured in interpretive notes and discussed in an interpretive team meeting; (2) the sentences and paragraphs were inductively analysed by one researcher to generate initial codes; (3) the codes were collected and categorized into potential themes, and all data relevant to each potential theme were collated and gathered; (4) the emerging themes were reviewed by another researcher who was not familiar with the transcripts to work in relation to the coded extracts and the entire data set; (5) interpretive writing on the ascertained themes was displayed; (6) clear definitions and names for each theme were analysed; and (7) report was produced.

### 2.4 Rigour

To establish reliability, several strategies were used according to relevant criteria,^27^ including credibility, dependability and transferability. Credibility was achieved by interviewing survivors of both sexes and across different age groups and areas of residence; in addition, the primary researcher performed the coding, categorisation and analysis to select the most suitable meaning unit. Dependability was achieved by peer evaluation of transcripts and group discussion to ensure that a consistent decision was made. Transferability was established by extensive clear and distinct descriptions of the culture and context, selection process and characteristics of the participants, data collection and the process of analysis. Meanwhile, we report the findings based on the COREQ checklist to ensure transferability.^28^

## 3 RESULTS

### 3.1 Demographic information

A total of 19 survivors with stroke were interviewed in this study, but none of them had taken part in the previous explorative interviews. The respondents' ages ranged from 42 to 89 years; 42.11% were female. Of these, 7 had a low risk of recurrent stroke and 12 had an intermediate risk of recurrent stroke. The duration of the interview ranged from 19 to 42 min, and a total of 94,000 words were transcribed. The characteristics of the participants are summarized in Table 1.

**Table 1:** Demographic characteristics (*N* = 19)

No.	Gender	Age (years)	Educational level	Diagnosis	NSE	ADL	Occupation	ESRS
S1	Female	60	Illiterate	IS & HS	1	Totally independent	Clean worker	0
S2	Male	66	Primary school	IS	1	Totally independent	Farmer	3
S3	Male	89	Primary school	IS	1	Totally independent	Worker	3
S4	Male	52	College	IS	1	Totally independent	Officer	1
S5	Female	73	College	IS	1	Totally independent	Farmer	3
S6	Male	66	High school	IS	1	Minimally dependent	Worker	5
S7	Male	53	Middle school	IS	2	Totally independent	Driver	2
S8	Female	72	Illiterate	HS	2	Minimally dependent	Farmer	3
S9	Male	78	College	IS	2	Minimally dependent	Worker	2
S10	Male	72	High school	IS	2	Minimally dependent	Teacher	3
S11	Female	58	Illiterate	IS	2	Partially dependent	Farmer	3
S12	Male	55	High school	IS	2	Partially dependent	Security	2
S13	Female	53	Illiterate	IS	3	Minimally dependent	Farmer	2
S14	Male	71	Middle school	IS	3	Partially dependent	Worker	4
S15	Male	72	High school	IS & HS	3	Partially dependent	Manager	4
S16	Male	66	Primary school	IS	4	Minimally dependent	Security	4
S17	Female	74	Illiterate	IS	4	Partially dependent	Farmer	4
S18	Female	42	Illiterate	IS	5	Minimally dependent	Farmer	2
S19	Female	67	Illiterate	IS & HS	7	Partially dependent	Farmer	3

*Note:* The ESRS is used to predict the risk of stroke recurrence; higher scores indicate higher recurrence risk. The risk is defined as low at 0–2, intermediate at 3–6 and high at 7–9. The Barthel index is used to assess stroke survivors' activities of daily living (ADL); 80–100 indicates total independence, 60–79 indicates minimal dependence, 40–59 indicates partial dependence and less than 40 indicates very high dependence on others.

Abbreviations: ESRS, Essen stroke risk score; HS, haemorrhage stroke; IS, ischaemic stroke; NSE, number of stroke events.

### 3.2 Perceptions of recurrence risk and behavioural changes

Nine major themes emerged, the themes with illustrative quotes are listed below, and examples of the illustrating coding tree are presented in the Supporting Information Appendix.

#### 3.2.1 First‐ever stroke survivors

##### Indifference to and unawareness of stroke recurrence risk

The first‐ever stroke survivors who had experienced mild stroke generally had complete confidence in their stroke prognosis and had no awareness of recurrence risk. A participant with a college degree stated, *‘*I have been here (hospital) two days, and I am very well, I don't think it (recurrence) will happen’ (S4). However, he was at low risk of stroke recurrence, according to ESER, and we found that he was reluctant to talk about the possibility of a relapse. Regardless of the educational background, an illiterate participant explained, ‘I got an (unclear) injection, and everything is ok now; it's ok’ (S1). An older man (S2) with primary school education also said, ‘This was just a minor cerebral infarction; it was nothing at all. My son has cerebral infarction too, and he is well now’. In terms of recurrence risk, some of the survivors had no idea of and never considered their risk of recurrence; as one participant asked, ‘what is recurrence and why? I am fine now’ (S1). Some survivors had complete confidence in their ability to prevent recurrence: ‘I have a good mentality and a high‐level adherence so it (recurrence accident) won't happen’ (S5); however, her Essen score was 3, indicating that she had an intermediate risk of stroke recurrence. Therefore, some survivors lacked knowledge of recurrence, some underestimated their susceptibility to and severity of stroke recurrence and some were unable or unwilling to make a connection between their disease and the possibility of recurrence (S3 and S4 avoided discussion of the topic of recurrence).

##### Need for professional support for information

Most first‐ever stroke survivors broadly mentioned warning signs when asked to recall the onset of stroke. Three survivors reported that they were able to identify anomalies quickly, but lacked accurate knowledge about the warning signs of stroke (S1, S2, S4). As S1 noted, ‘I do not know what happened, but I can feel that one of my legs is weak’.‘I felt weakness suddenly, I did not know exactly what happened, my son took me to the hospital’ (S2). However, there were patients who ignored the significance of emergency care because they were unaware of the symptoms; one patient who experienced a TIA before stroke did not know about the TIA at all: ‘I did not know what happened at that time. I couldn't remember the falling accident, so I did not tell them (his wife and daughter)’ (S6). Although two interviewees had a positive learning attitude and they could recognize stroke once they were aware that they suffered a stroke, they gained related knowledge from others, the TV or books, rather than from medical staff, as one older man noted, ‘I always saw some people with hemiplegic paralysis walked around in the garden. I asked them, and then I knew some warning signs of stroke’ (S3). Another patient mentioned, ‘I like to read books and watch TV. So, I know some; that is why I was able to visit the hospital in time’ (S5, she laughed and was proud). Both participants mentioned knowing ‘some…’; this might indicate the fact that they still need professional support to obtain information.

##### Different awareness of the importance of different healthy behaviours

Participants' treatment‐seeking behaviour was relatively active; they knew that it was necessary to visit the hospital if they experienced any symptoms. As some participants highlighted, *‘*At nine o'clock pm, I felt uncomfortable and could not move my leg, I did not know what happened… I told my son, he called 120 (emergency hotline) and sent me to this hospital immediately’ (S1). However, when talking about secondary prevention behaviour, most of them were aware of the importance of medication intake, but some of them were  not aware of the importance of lifestyle modifications, as emphasized by S3, ‘It is critical to adhere to physicians' advice to take medication’. On probing patients' perceptions, most participants reported feeling guilty about their previous self‐medication behaviour; ‘I threw away the medicines after being diagnosed with atrial fibrillation, it was boring, I won't do again…’ said S1 with regret. In terms of lifestyle modifications, many showed an indifferent attitude, as S2 claimed: ‘Many people around me suffered stroke, but they still had to work (earn money), who care about anything else? I have to earn money; I am fine now…’ Moreover, some patients who lived in an extended family set‐up had limited freedom to choose what to eat ‘I cannot, they (son or daughter‐in‐law) cooks and I eat, we have no awareness of “healthy food”’ (S1).

#### 3.2.2 Survivors with a second stroke

##### Worry, but feel powerlessness towards recurrent event

We found that all the participants expressed different degrees of worry or fear about their prognosis. Patient S7, a middle‐aged male driver, explained, ‘I am afraid; although I'm afraid, I do not know what to do at all…’. A female stroke patient (S8) also noted, ‘I'm afraid. I'm afraid to come back here (hospital) again’. The decline in self‐care ability caused by the disease also directly led to a sense of powerlessness, as some of them explained: ‘Another cerebral infarction, what is a big deal? What else can you do? What can you do (nothing…)?’ (S7). Patient S9 reported, ‘You cannot do anything at home. I am getting old. I have no thoughts of future’. One of the participants wanted to find a job and return to work, but he was rejected: ‘I tried to find a job, but they finally sent me back to home, I felt weak, and I could not walk a long distance’ (S10). The occurrence of recurrence events, worsening of symptoms after recurrence, lack of knowledge about the prognosis of recurrent events and sense of powerlessness may be the reasons that led to the interviewees' negative emotional responses.

##### Support in terms of obtaining accurate information is still warranted

The lack of accurate information about stroke was still an unresolved concern among recurrent survivors. As one middle‐aged patient (S7) explained, ‘I felt uncomfortable and dizzy; I thought it might be another stroke’. S8 stated, ‘I never thought that it was a recurrent stroke; I thought I might have caught a cold, but it progressed quickly’. S11 claimed, with a smile, ‘I thought the doctor scared me when he told me the recurrence risk; I did not take it seriously’. Receipt of inadequate or insufficient information led to lack of confidence among the survivors. One patient described his feelings regarding his second stroke as follows: ‘I do not know what happened, this makes me unhappy and confused’ (S10). When the participants were asked how stroke can be prevented, most of them still could not answer correctly. Even though some of the participants quit smoking after their first stroke, they did not know why they had to change their behaviour; ‘they (doctor or nurse) always gave me some suggestions in a hurry, but I could not understand very well, and I didn't know why. They are so busy…, I don't know how to ask…’ S7 said and scratched his head. Thus, it can be seen that accurate and sufficient information is still needed. A middle‐aged male survivor (S12) stated that ‘Even though there are lots of health education flysheets in the hospital, but I never read them, I have no patience to read them…’.

##### Regret of unhealthy behaviour patterns

A recurrent event is possibly a triggering factor that could increase survivors' motivation to change unhealthy behaviours. Some of the participants expressed regret and described why they felt self‐reproach. One participant (S7) explained, ‘I did not think of it here; I did not take it seriously. I must be wrong’.‘I did not want to take medicine; I thought I was well, but now I'm in trouble (scratch head, sigh, etc)’. Poor adherence to medication was considered to be the main reason for recurrence, as illustrated by the following statement: ‘I did not take the medication according to the prescription, but I should do’ (S8). In addition to medication adherence, poor compliance with functional exercises was mentioned as a barrier; a middle‐aged man (S10) noted: ‘No one told me how to exercise after discharge, and I failed to do rehabilitation exercises every day’. Some survivors also regretted losing their temper: ‘you cannot get angry, this time, I was angry with my children, so it happened again…, I knew I shouldn't, but I couldn't control myself…’ (S9). ‘The impact of getting sick was great, and people who have not had it do not know this kind of pain; I cannot bear the burden of my thoughts anymore’ (S10). While making this statement, it was observed that the interviewee was trying to control his emotions. Most participants had some degree of regret of incompliance or unhealthy behaviours.

#### 3.2.3 Survivors with multiple recurrences

##### Perceived severity of recurrences

All participants reported that they perceived the severity of the stroke, and they noted participation limitations caused by frequent stroke events. As one participant explained, ‘The first time, I was young, and I did not pay attention to it. The second time, I could not speak very clearly, but I recovered within two weeks. This time, I cannot do housework; I think it must be much worse…’ (S13). ‘The first time was just a case; the second time, it had a relatively mild impact on my life. However, this time it was too heavy, eh!’ (stated by S14). ‘I was able to go out and go around before, but I have urinary incontinence this time; I will not do anything outside, as I am a little ashamed and it is inconvenient’, as stated by S16, who used to be a security guard. ‘I cannot do anything, I liked square dancing, but now, it's impossible…’ claimed by S18. Limitation in terms of social participation is a common problem among stroke survivors with severe physical disabilities or communication disorders.

##### Need for increased psychological care

A belief in fatalism was expressed and seemed to affect stroke survivors' attitudes towards their disease.^29^ Some participants held ambivalent attitudes; on the one hand, they thought that the disease was a natural process, there was nothing they can do to prevent or stop it (recurrence) from happening and people will get sick and die anyway: ‘Death. Nothing can be done to prevent death. I won't hide, and I know I cannot change the process; each person will die’ (S13). On the other hand, they showed a significantly passive attitude towards stroke recurrence; four participants recognized their stroke recurrence as uncontrollable and unpreventable; a strong sense of failure and powerlessness was evident from their words: ‘When I met some old friends in the garden, I smiled, and we did not talk with each other as we know what will happen next’ (S15). ‘I cannot get around, and I don't like to do things; I just want to sit here and wait’ (S17). ‘I do not want to do anything; whatever, it (recurrence) will occur’ (S18), and ‘It is impossible to worry, and I never think about it’ (S19). An older man said, ‘someone told me I would die at 70, and now I only have four years left. I want to do what I like (drinking and eating my favourite food)’ (S16). We could observe that it was difficult for the participants who had lost confidence in terms of control over their life to talk about this topic, which indicates that they might need more psychological care.

##### Incorrect perceptions of healthy behaviours

Perceived benefit is a critical motivator for chronic disease survivors to change their behaviour.^29^, ^30^ Behavioural change is beneficial only when the survivors know what healthy behaviour is and what benefits they can gain. However, no perceived visible benefit is a barrier in this study. As one male stroke patient with a moderate level of dependence in terms of daily activities said, ‘I am doing exercise every day, but I am sick again; It may be too much exercise, who knows why it happened again and again?’ (S14). One patient (S16) mistakenly thought that alcohol could reduce blood sugar levels; he explained, ‘I think alcohol cessation was good for controlling blood sugar. However, it seemed that drinking some alcohol effectively reduced my blood sugar level; who knows why. Whatever; I started to drink again’. A female patient (S17) with self‐perceived high‐level adherence suffered stroke four times. She expressed doubts about the effects of treatment and healthy behaviours: ‘I never forgot to take my medication, and I paid attention to diet, exercise, etc., but it occurred again. What is going on?’ (The interviewee showed irritability and had a helpless smile). Another patient who exercised every day noted, ‘I do not know how to exercise; I just do as much exercise as I can, if I have time’ (S14). Another patient with ‘high‐level’ adherence changed her medication because it was too expensive (S17).

## 4 DISCUSSION

In extending previous quantitative evidence on this topic, this study provides additional insight into participants' feelings and attitudes towards recurrence risk and behavioural changes. This analysis uncovered nine major themes regarding first‐ever or recurrent stroke survivors' experiences and feelings. Participants with first‐ever stroke tended to ignore or are unaware of recurrence risk, while recurrent events led to awareness of the severity of recurrence, but made them feel powerless. Moreover, survivors with multiple relapses showed a triple attitude toward recurrence risk, e.g., negative acceptance, positive acceptance, or both, which indicate an increased psychological care need. Also, their attitudes toward behavioural changes were influenced by the number of relapses as well, in terms of the decision made to make changes in their life, regret for nonadherence to medication and questioning the benefits of behavioural changes.

As Hiraga^7^ commented, knowledge and perception of recurrent stroke risks and risk factors are very important from the points of view of education and secondary prevention strategies. However, in these interviews, it was found that almost all survivors had only recently heard of the risk of recurrence and had limited knowledge about the likelihood of recurrence. Still, they rarely considered the possibility of susceptibility to recurrence; this result could be related to the Chinese cultural context, as people prefer to adopt an optimistic attitude and discuss positive outcomes.^31^ Although the findings of this study were based on interviews with only a few individuals, previous studies in different countries and regions have reported similar results.^10^, ^12^, ^32^, ^33^ Boden et al.^12^ investigated the perception and awareness of recurrence risk in survivors with ischaemic stroke and transient ischaemic attack. The results revealed that only 20% accurately perceived the risk of recurrence; 10% of these patients underestimated the risk of recurrence and 70% of the survivors overestimated the risk. Another study conducted by Croquelois and Bogousslavsky^11^ reported the perceived risk of recurrence among survivors with first stroke after 3 months; 65.2% of survivors claimed that they did not consider the risk of recurrence (e.g., recurrence should not happen to me). Similarly, a study conducted in a developing country determined and compared the self‐perceived risk of recurrence and the actual risk of recurrence in survivors with transient cerebral ischaemia and found that most survivors did not accurately perceive their risk of recurrent stroke; 43.6% of the survivors underestimated the risk and 17.1% of the survivors overestimated the risk.^10^ It can be seen that poor perception of recurrence risk is a global problem, and an individual's perception of recurrence risk is likely to have a strong impact on their prognosis and long‐term outcomes.

We also found that the unmet need for medical information was a general problem among most participants in this study, especially in terms of warning signs and the treatment time window. Even in the case of a patient with a high level of education, when we asked him about the best treatment administration time, he claimed that it was within 12 h after onset (S6, Manager); furthermore, most of the remaining survivors could not accurately specify the proper treatment time. Previous studies have shown that only very few stroke victims (16.9%) recognize the initial signs of stroke; not only stroke survivors but also their family members need support in terms of receiving information on stroke.^34^ Even in developed countries, the most common (73.8%) unmet need of stroke survivors in the acute stage is stroke education.^35^ In addition to the survivors with first‐ever stroke, the survivors with multiple recurrences had no confidence in their ability to identify or recognize the initial signs of stroke accurately. Thus, they were able to recognize slight abnormalities and seek medical treatment as soon as possible. However, in most cases, it is not the patients, but their children or spouses who called the ambulance service. In addition, when survivors with stroke lack sufficient information, they may lose confidence and motivation to make behavioural changes in response to recurrences and engage in rehabilitation.^36^, ^37^ Lack of knowledge can become an obstacle to behavioural changes if survivors cannot perceive the benefits as well, as described by some interviewees. Survivors were willing to make additional changes if the changes were perceived to be beneficial to their health.^38^ Therefore, despite extensive efforts, further dissemination of information of and education about the benefits of lifestyle modifications are still needed worldwide.

Additionally, our findings suggested that participants with or without recurrence have different attitudes towards behavioural changes. We found that almost all the participants were prompt in quickly going to the hospital following the onset of symptoms, even though most of them failed to precisely describe the warning signs or the time window for the treatment of stroke. For the first‐ever stroke survivors, this was due largely to their family members in this study, as S1/S2/S3 mentioned, which was consistent with the literature that having a knowledgeable bystander was associated with appropriate help‐seeking behaviour and shorter prehospital delay.^39^ Although previous studies have shown that inability to accurately identify the symptoms of stroke and failure to treat stroke as an emergency can result in longer prehospital delay,^40^ awareness of the severity can also promote appropriate help‐seeking and early arrival at a stroke centre.^39^ For the participants with recurrent stroke, it has been argued that perception of severity of and susceptibility to stroke may be trigger factors for behavioural modifications.^8^, ^39^ However, in terms of self‐perception of the risk of disease, underestimation of risk or ‘optimism’ may be a barrier to the adoption of preventive health behaviours as well.^41^ We found that patients with a second incidence of stroke adopted healthy behaviours but they also complained about lack of knowledge and feeling powerless. Also, some patients blamed their recurrence on fate, as an excuse of his/her maladaptive process^29^; this is also in agreement with our findings among patients with multiple recurrent events. We found that patients with multiple recurrences were sceptical about healthy behaviours, and they were more inclined to indulge themselves rather than strictly manage their behaviour. This may be primarily because patients cannot perceive any benefits from changing their behaviour in the short term, which is particularly important, as considerable effort is involved in changing these behaviours. Indeed, the benefits of behavioural changes are complex, dynamic and could be cumulative.^42^, ^43^

These findings provide us with a better understanding that accurate information and education about perceived benefits are necessary to promote active responses among survivors of stroke and facilitate healthy behaviours.^7^ Accurate information about warning signs, onset of symptoms and other disease‐related information should be provided, and proper education to raise awareness of the benefits of healthy behaviours to prevent stroke recurrence may be more appealing and acceptable to stroke survivors with multiple recurrences. Considering misperceptions of disease risk are common among public,^19^ strengthened education on recurrence risk communication could be a possible alternative to resolve this problem.^44^, ^45^ It can help stroke survivors realize that they are a high‐risk group for recurrence, and they need to use proactive strategies to prevent it.^40^ The most common risk communication programmes among stroke survivors mainly focus on treatment decisions for intravenous thrombolysis or other medical treatments.^40^, ^46^ However, few studies have focused on the effect of recurrence risk assessment and standardized risk communication tools among stroke survivors. Therefore, relevant risk communication education should be provided to improve stroke survivors' perception of recurrence risk. Additionally, the findings of this study also highlighted that professionals should explore more targeted interventions in the future based on the number of relapses that patients experience.

### 4.1 Strengths and limitations

This study delineates typical perceptions according to the number of recurrences of stroke. However, this study is not without its limitations. First, the participants with first‐ever or recurrent stroke in this study were mildly or moderately dependent on others for daily activities, and we mainly included low‐ and intermediate‐risk survivors, and although the generalisability of these study findings was limited, their perceptions of recurrence risk were suboptimal, and these outcomes were similar. Second, we only recruited stroke survivors without problems in communication to obtain more useful information; the perception of recurrence risk among a broader study population needs to be explored for the development of tailored interventions. Furthermore, we attempted to discuss the changes in recurrence risk perception among stroke survivors with different recurrence times, and it would be better to conduct a longitudinal qualitative study on a fixed sample to explore the complex and dynamic nature of recurrence risk perception over time.

## 5 CONCLUSIONS

This study found that even survivors with multiple incidences of stroke do not have a good understanding of the risk of recurrence. The lack of awareness of the risk of recurrence after the first attack, the worry caused by the first recurrence and the inability to cope with the recurrence and positive or negative acceptance of multiple recurrences provide further evidence of the need for support in terms of information provision. It is necessary to develop a risk communication education plan during hospitalisation for stroke survivors who have experienced multiple incidences of stroke and to highlight the importance of the required information according to the needs of the survivors as much as possible. This approach could improve information acceptance, understanding and mastery among stroke survivors and their families and could ultimately promote health‐promoting behaviours and long‐term recovery.

## CONFLICT OF INTERESTS

The authors declare that there are no conflict of interests.

## AUTHOR CONTRIBUTIONS

Bei‐lei Lin conceptualized the study. Bei‐lei Lin and Yun‐fei Guo developed the interview guide, and Zhen‐xiang Zhang approved the guideline. Bei‐lei Lin and Yun‐fei Guo collected the data, supported by Yong‐xia Mei and Wen‐na Wang. Bei‐lei Lin, Yun‐fei Guo and Wen‐na Wang transcribed the interviews and analysed and interpreted the data. Bei‐lei Lin wrote the first draft of the manuscript. Yong‐xia Mei and Yao Tong verified the initial translation of code. Daphne Cheung, Nazia Shuaib and Shan‐shan Wang reviewed the translation. Zhen‐xiang Zhang and Daphne Cheung performed the quality control of this study, and Daphne Cheung revised the manuscript critically for important intellectual content. All authors have read and approved the final manuscript.

## ETHICS STATEMENT

Ethical approval was obtained from the Ethics Committee (ZZURIB2019‐005), and approval for data collection was obtained from the Science and Research Department of the three hospitals.

## Supporting information

Supporting information.

Supporting information.

## Data Availability

The data sets analysed during the current study are available from the corresponding author on reasonable request.
