# Supplementary material for: Perceptions of recurrence risk and behavioural changes among first‐ever and recurrent stroke survivors: A qualitative analysis
Source: Health Expect. 2021 Aug 6;24(6):1962–70. doi: 10.1111/hex.13335 (PMC8628583; doi:10.1111/hex.13335)
Supplement: Supplementary file 1 — Supporting information. [file HEX-24-1962-s002.docx]

**Appendix**

**Interview guide**

1. What was your experience of stroke when it happened to you?
2. What do/did you think about disease for you and your family?
3. What had changed (will) in your life before and after the illness (description of a typical daily life before and/or after the illness)?
4. What do you think about your recurrence risk? (or What is your feeling about recurrent stroke? )
5. How do you think about the significance and value of healthy behaviours (lifestyle)?
6. What are the barriers and facilitators you see when deciding to change (not change)/ when you change unhealthy behaviour?
