# Supplementary material for: Perceptions of recurrence risk and behavioural changes among first‐ever and recurrent stroke survivors: A qualitative analysis
Source: Health Expect. 2021 Aug 6;24(6):1962–70. doi: 10.1111/hex.13335 (PMC8628583; doi:10.1111/hex.13335)
Supplement: Supplementary file 2 — Supporting information. [file HEX-24-1962-s001.docx]

**Appendix**

**Themes, codes and illustrating quotations**

| **Themes** | | **Codes** | **Illustrative quotes** |
| --- | --- | --- | --- |
| **First-ever stroke survivors** | |  |  |
|  | Indifference and unawareness of stroke recurrence risk |  |  |
|  |  | - recurrence won’t happen - no awareness of recurrence | - A participant with a college degree reassured and said, “*I have been here (hospital) two days, and I am very well, I don’t think it (recurrence) will happen*.” (S4) - *“…what is recurrence and why? I am fine now...it’s not a big deal, I am healthy, and I like do exercise, it won’t happen, I think…”* (S4) |
|  |  | - no awareness of recurrence risk | - A female participant with elementary school education smiled and said, “*When my son took me to this hospital, I got an injection, I don’t know what it is, but everything is ok now, you see…I am well enough to be discharged from the hospital, it’s ok…I think…*” (S1) |
|  |  | - stroke is a common disease - stroke can be easily prevented | - An older man with primary school education said with an indifferent tone, *“This was just a minor cerebral infarction… it was nothing at all. My son has cerebral infarction too, and he is well now.”* - *“…if you don’t want it (stroke or recurrent stroke) happen, taking regular annual intravenous infusions are sufficient, we have a village doctor, you don’t even have to take oral medication… I think, also, my son thinks so…”* (S2) |
|  |  | - have the confidence to prevent a recurrence | - A female participant with a college degree said with complete confidence*, “I have a good mentality and a high-level adherence so it (recurrent accident) won’t happen, I always read relevant books or watch healthy related TV programs, so I know how to prevent it…”* (S5) |
|  |  | - reluctant to talk about recurrence | - *A senior stroke patient said, “I never forget to take medication, and I go for a walk every day, I have to take care of myself, they (children) are busy, so I don’t want talk about…(recurrence)”* (S3) |
|  | Professional information support need |  |  |
|  |  | - have no idea about the warning signs of stroke | - “*I did not know what happened, but I felt that one of my legs was weak on that night, I told my son, and he said ‘let’s go to hospital…’, he might know what happened, my husband was a stroke patient, I had no idea, just felt weakness..*.” (S1 said with a smile) |
|  |  | - have no idea about the warning signs of stroke | - “*I felt weakness suddenly, and I did not know what happen, my son took me to here (the hospital) ...*” (S2 said with an anxious tone because he wanted to go back to work) |
|  |  | - have no awareness of TIA - did not tell family members | - S6 recalled, *"I did not know what happened at that time. I couldn’t remember the falling accident, so I did not tell them (his wife and daughter)." His wife said she didn’t know TIA as well, and she did not know her husband used to have TIA.* - *Then S6 said, “…this time they (spouse and daughter) took me to the emergency department…”* (S6 said with indifferent tone) |
|  |  | - obtain information from other patients | - *“I always saw some people with hemiplegic paralysis walked around in the garden. I asked them, and then I knew* ***some*** *warning signs of stroke…”* (S3 smiled and said) |
|  |  | - obtain information from books, TV, etc. | - *"I like to read books and watch TV. So, I know* ***some****; that is why I was able to visit the hospital in time.”* (S5 said with proud tone) |
|  | Different awareness of importance of different healthy behavior |  |  |
|  |  | - active treatment-seeking behavior | - *“At nine o’clock pm, I felt uncomfortable and could not move my leg, I did not know what happened… I told my son, he called 120 (emergency hotline) and sent me to this hospital immediately"* (S1) |
|  |  | - pay attention to medication-taking | - *“It is critical to adhere to physicians’ advice to take medication, it’s very essential, and if you feel sick, just visit the hospital quickly, the doctors here know my situation very well, we are friends, I have medical insurance, following their guidance is critical..*.” (S3) |
|  |  | - regret not taking the medication regularly | - “*I threw away the medicines after being diagnosed with atrial fibrillation, it was boring*, *I wouldn’t do again*…” *It’s expensive to take pills every day, I can’t earn money, my husband had a stroke, this is why I couldn't take medicines regularly…* *but now, we spent more money this time…*” (S1) |
|  |  | - can’t decide what to eat, and don’t know what healthy food is | - *“I cannot, they (son or daughter-in-law) cook and I eat, I never cook by myself, they decide what to eat, and we have no awareness of ‘healthy food*’.” (S1) |
|  |  | - have no time to consider lifestyle modification | - “*Many people around me suffered a stroke, but they still had to work (earn money); who care about anything else*? *I have to earn money…we have no time to think about this, we have to earn money, then how to change lifestyle?* *I eat at construction sites and have to work every day, so it is impossible…now, I think to earn money is more important for (me or my family)”* (S2) |
| **Survivors with a second stroke** | |  |  |
|  | Worry but feel powerlessness towards the recurrent event |  |  |
|  |  | - inability to cope with recurrence - fear of recurrence | - *"Another cerebral infarction, what is a big deal? What else can you do? What can you do (nothing…)?"* (S7) - *“I am afraid; of course, I am worried, although I'm afraid, I do not know what to do at all…* " (S7 smiled and scratched his head, he kept on saying he was afraid) |
|  |  | - fear of recurrence - uncertainty about the future | - *“The doctor told me that I could go home next Monday, … but I'm afraid, I dared not leave the hospital. I'm afraid to come back here (hospital) again…”* - *“I never thought the disease would happen, I didn’t believe it did happen…, but now, I am scared…”* (S8) |
|  |  | - powerlessness about the future | - *"You cannot do anything at home. I am getting old. I have no thoughts of future, no one can stop disease... "*(S9) |
|  |  | - powerlessness about the future | - *" I tried to find a job, but they finally sent me back to home, I felt weak, and I could not walk a long distance, so I cannot do any job...now…”* (S10) |
|  | Accurate information is still warranted |  |  |
|  |  | - need secondary prevention information - health education is not sufficient | - “*I cannot do anything, except for taking medication, eating… just pay a little attention to it…there is no use to pay attention to it…I think… taking medication is more important and useful…*” (S7) - “*They (doctor or nurse) always gave me some suggestions in a hurry, but I could not understand very well, and I didn’t know why. They are so busy…, I don’t know how to ask…*” (S7) |
|  |  | - guess but not sure what happened | - *“After the first stroke, I thought that I could return to work, but when I was driving, I felt uncomfortable and dizzy suddenly; I thought it might be another stroke, although I was not sure, I guessed it might be stroke again...”* (S7) |
|  |  | - cannot recognize stroke warning signs | - *“I never thought that it was a recurrent stroke; I was sleeping; initially, I thought I might have caught a cold, but it progressed quickly, I could not lift my hand, I called my husband, I never thought it was stroke...before I came here…”* (S8) |
|  |  | - not paying attention to recurrence | - *“I thought the doctor scared me when he told me the recurrence risk; I did not take it seriously. But it did happen…”* (S11 claimed with a smile) |
|  |  | - need more individualized information support | - *“Even though there are lots of health education flysheets in the hospital, but I never read them, I have no patience to read them…they (doctors) just asked me to do rehabilitation, but they did not give me a detailed and clear rehabilitation plan, I want to know more…about how to return to work (or normal life) …”* (S12) |
|  | Regret of unhealthy behavior |  |  |
|  |  | - regret not paying attention - regret not following discharge guides | - *"I did not think of it (recurrent stroke) here; I did not take it seriously. I must be wrong."* - *"I did not want to take medicine; I thought I was well…and I went to work…I am a driver, I have to drive the car at night…So, I have no time to adjust my eating habit…sometimes, I drunk, but only a little… now I'm in trouble (scratch head, sigh, et c.). "*(S7) |
|  |  | - not taking medicines according to prescription - decide to adhere to prescription after discharge | - *“I did not take the medication according to the prescription, but I should do; I am taking care of my daughter’s baby recently, she is very busy, I almost forget that I used to be a patient, so…it happened again.”* - *“I have to pay attention to this (disease) in future, because I have to help my children…”* (S8). |
|  |  | - regret not managing temper | - “*You cannot get angry, this time, I was angry with my children, so it (stroke) happened again…, I knew I shouldn’t lose temper, but I couldn’t control myself…*” (S9) |
|  |  | - attributing the problem to lack of exercise - distressed emotion | - *"No one told me how to exercise after discharge, I failed to do rehabilitation exercises every day.... I thought this was the main reason, if they could give me an individualized or detailed rehabilitation plan, recurrent stroke would not happen…I don’t know who I can ask…they (doctors) are busy, besides, after discharge, I don’t know who else I can phone…”* - *"The impact of getting sick was great, and people who have not had it do not know this kind of pain; I cannot bear the burden of my thoughts anymore…"* (S10) |
| **Survivors with multiple recurrences** | |  |  |
|  | Perceived severity of recurrences |  |  |
|  |  | - decreased ability to perform daily life due to recurrences | - *"The first time, I was young, and I ignored it. The second time, I could not speak very clearly, but I recovered within two weeks. This time, I cannot do housework; I think it must be much worse...I never thought it could happen again and again…what’s the reason?"* (S13) |
|  |  | - perceived severity of recurrences | - *"The first time was just a case; the second time, it had a relatively mild impact on my life. However, this time it was too heavy, eh!"* (sighed by S14) |
|  |  | - physical disability due to recurrences | - *" I was able to go out and go around before, but I have urinary incontinence this time; I will not do anything outside, as I am a little ashamed and it is inconvenient."* (highlighted by S16) |
|  |  | - decreased social participation ability due to recurrences | - *“I cannot do anything, I liked square dancing, but now, it’s impossible…I am young, I don’t know why this happened to me, I even don’t know what I can do, there are so many works need to be done…my children are still in school…”* (S18) |
|  | Increased psychological care need |  |  |
|  |  | - actively accept the possible outcomes caused by stroke - have no ability to prevent (recurrent outcomes) | - *"Death, we cannot do anything to prevent death. I won’t hide…”* - *I know I cannot change the process; each person will die... my father died of stroke, he always told me that everyone would die, just a matter of time”* (S13) |
|  |  | - passively acceptance | - *“When I met some old friends in the garden, I smiled, and we did not talk with each other as we know what will happen next…no one knows what to say, it seemed like ‘It's going to rain and people are going to die’, everything is destiny…so I have to accept it”* (S15). |
|  |  | - have to watch TV every day - passively stand by and watch | - *“I cannot get around, and I don’t like to do things; I just want to sit here and wait…I watch TV every day, or I spent most of the time watching TV, I don’t want to change, there is no use to change, who knows when it (recurrence or death) will come next time?”* (S17). |
|  |  | - feel powerless to prevent recurrent stroke | - *“I do not want to do anything; whatever, it (recurrence) will occur”* (S18) |
|  |  | - believe in fate and wait passively | - *“Someone told me I would die at 70, and now I only have four years left. I want to do what I like (drinking and eating my favourite food)” (S16)* |
|  |  | - avoid discussing this issue (future) | - *“It is impossible to worry, and I never think about it”* (S19) |
|  | Incorrect perceptions of healthy behavior |  |  |
|  |  | - question the benefits of exercise | - *"I am doing exercise every day, but I am sick again; It may be too much exercise, who knows why it happens again and again?"* (S14) |
|  |  | - not experiencing the benefits of sobriety | - *"I think alcohol cessation was good for controlling blood sugar. However, it seemed that drinking some alcohol effectively reduced my blood sugar level; who knows why. Whatever, I started to drink again.” (S16)* |
|  |  | - question the treatment effect of medication | - *“I never forgot to take my medication, and I paid attention to diet, exercise, etc., but it occurred again. What is going on?"* (Interviewee showed irritability and a helpless smile) (S17) |
|  |  | - incorrect exercise habit | - *“I do not know how to exercise; I just do as much exercise as I can, if I have time”* (S14) |
|  |  | - self-medication behavior | - Another patient with “high-level” adherence changed her medication because it was too expensive (S17) |
